# Inflammatory Signalings Involved in Airway and Pulmonary Diseases

**DOI:** 10.1155/2013/791231

**Published:** 2013-04-04

**Authors:** I-Ta Lee, Chuen-Mao Yang

**Affiliations:** ^1^Department of Anesthetics, Chang Gung Memorial Hospital at Linkuo and Chang Gung University, Kwei-San, Tao-Yuan 33302, Taiwan; ^2^Department of Physiology and Pharmacology and Health Aging Research Center, College of Medicine, Chang Gung University Kwei-San, Tao-Yuan 33302, Taiwan

## Abstract

In respiratory diseases, there is an increased expression of multiple inflammatory proteins in the respiratory tract, including cytokines, chemokines, and adhesion molecules. Chemokines have been shown to regulate inflammation and immune cell differentiation. Moreover, many of the known inflammatory target proteins, such as matrix metalloproteinase-9 (MMP-9), intercellular adhesion molecule-1 (ICAM-1), vascular cell adhesion molecule-1 (VCAM-1), cyclooxygenase-2 (COX-2), and cytosolic phospholipase A_2_ (cPLA_2_), are associated with airway and lung inflammation in response to various stimuli. Injuriously environmental stimuli can access the lung through either the airways or the pulmonary and systemic circulations. The time course and intensity of responses by resident and circulating cells may be regulated by various inflammatory signalings, including Src family kinases (SFKs), protein kinase C (PKC), growth factor tyrosine kinase receptors, nicotinamide adenine dinucleotide phosphate (NADPH)/reactive oxygen species (ROS), PI3K/Akt, MAPKs, nuclear factor-kappa B (NF-**κ**B), activator protein-1 (AP-1), and other signaling molecules. These signaling molecules regulate both key inflammatory signaling transduction pathways and target proteins involved in airway and lung inflammation. Here, we discuss the mechanisms involved in the expression of inflammatory target proteins associated with the respiratory diseases. Knowledge of the mechanisms of inflammation regulation could lead to the pharmacological manipulation of anti-inflammatory drugs in the respiratory diseases.

## 1. Introduction

Inflammation is a protective response to cellular and tissue damage/injury. The purpose of this process is to destroy and remove the injurious agents and injured tissues, thereby promoting tissue repair. When this beneficial response occurs in an uncontrolled manner, the result is excessive cellular/tissue damage that results in chronic inflammation and destruction of normal tissue [1]. Moreover, inflammatory airway and lung diseases, such as asthma or chronic obstructive pulmonary disease (COPD), are characterized by chronic inflammation. Many of the known inflammatory target proteins, such as matrix metalloproteinase-9 (MMP-9), intercellular adhesion molecule-1 (ICAM-1), vascular cell adhesion molecule-1 (VCAM-1), cyclooxygenase-2 (COX-2), and cytosolic phospholipase A_2_ (cPLA_2_), are associated with inflammatory signaling pathways induced by various stimuli, including tumor necrosis factor-*α* (TNF-*α*), interleukin-1*β* (IL-1*β*), adenosine-5′-triphosphate (ATP), cigarette smoke extract (CSE), lipoteichoic acid (LTA), or lipopolysaccharide (LPS) [2–6]. Airway smooth muscle is considered as an end-response effector regulating regional differences in ventilation by contracting in response to various proinflammatory mediators and exogenous substances released under homeostatic or pathologic conditions, such as asthma [7]. Lung cells, in particular alveolar epithelial type II cells, are susceptible to the injurious effects of oxidants. It has been shown that lung cells release inflammatory mediators and cytokines/chemokines, such as IL-1*β*, IL-6, IL-8, and TNF-*α* in response to oxidative stress [8]. Moreover, the SFKs, PKC, growth factor tyrosine kinase receptors, NADPH oxidase/ROS, PI3K/Akt, and MAPKs are components of signaling cascades that respond to extracellular stimuli by targeting transcription factors, such as NF-*κ*B and AP-1, resulting in the modulation of inflammatory gene expression [8]. Thus, this review will focus on some general aspects of inflammatory signaling regulation and summarize current knowledge regarding the presence and functional roles of these inflammatory signal molecules within the respiratory system, and their proposed involvement in the expression inflammatory target proteins in response to proinflammatory mediators during airway and lung inflammation. The pharmacological interventions protect against inflammation-induced airway and lung diseases will be discussed.

## 2. Inflammatory Target Proteins and Respiratory Diseases 

### 2.1. Adhesion** **Molecules

**Figure 1:**
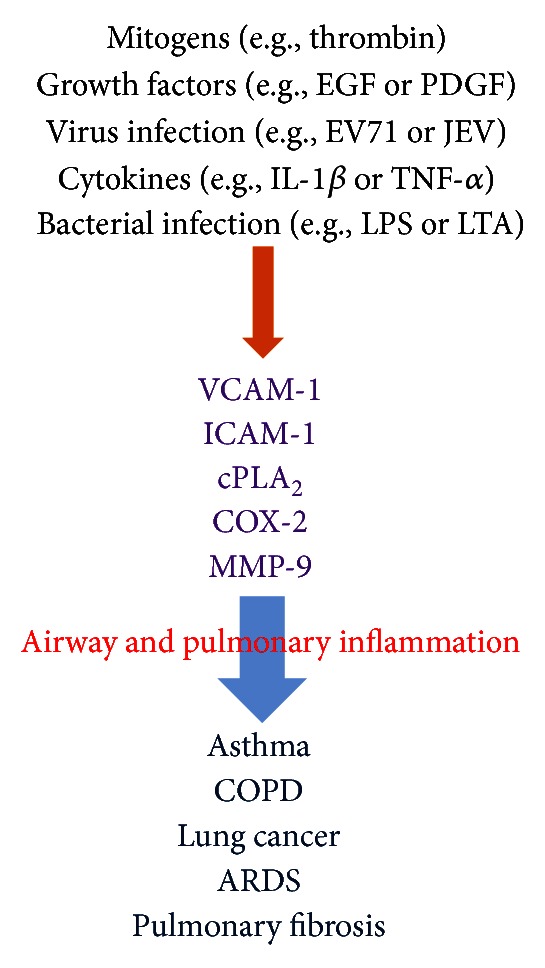
General overview of the inflammatory target proteins in respiratory diseases. Many of the known inflammatory target proteins, such as MMP-9, ICAM-1, VCAM-1, COX-2, and cPLA_2_, are induced by various stimuli, such as mitogens, growth factors, virus infection, cytokines, and bacterial infection. EGF, epidermal growth factor; PDGF, platelet derived growth factor; EV71, enterovirus 71; JEV, Japanese encephalitis virus; IL-1*β*, interleukin-1*β*; TNF-*α*, tumor necrosis factor-*α*; LPS, lipopolysaccharide; LTA, lipoteichoic acid.

Cell adhesion molecules play an important role in inflammatory responses. Leukocytes continuously circulate throughout the body in order to come in contact with antigens sequestered within tissues. To enter tissues, circulating leukocytes migrate from the blood, between vascular endothelial cells and into the tissue [9]. During this migration, leukocytes initially bind to endothelial cells via low affinity adhesion molecules. The low affinity adhesion in combination with the force of the blood flow results in rolling of leukocytes on endothelial cells. Subsequently, adhesion molecule affinity is upregulated and leukocytes firmly adhere to the endothelium [9]. Finally, bound leukocytes migrate between the endothelial cells and into the tissue. VCAM-1 is one of the inducible cell transmembrane glycoproteins of the immunoglobulin supergene family expressed on several cell types and plays an important role in a number of inflammatory and immune responses [10]. It was first identified as an adhesion molecule induced on endothelial cells by proinflammatory cytokines or LPS [11, 12]. In normal processes, VCAM-1 is important during development since a VCAM-1 knockout is an embryonic lethal. In pathogenesis, VCAM-1 expression is induced on endothelial cells during inflammatory bowel disease, atherosclerosis, infection, and asthmatic responses [13–15]. Upregulation of VCAM-1 expression on cytokine-triggered vascular endothelial cells enhances the targeted transmigration of PMNs into extravascular space of inflammation [10]. In airways, to reach the submucosa and airway lumen, circulating PMNs must first be recruited across the vascular endothelium [16] and then migrate through the interstitial matrix before interacting with airway epithelium. The pathogenesis of asthma, eosinophil migration into the lung is VCAM-1 dependent [17]. Accumulation of inflammatory cells within the airways can be influenced by expression of adhesion molecules on airway epithelium. Thus, similar processes that govern PMNs adhesion to lung airway resident cells may occur and contribute to the damage to these cells seen in inflammatory responses of asthma [18]. This event is crucial in the development of allergic inflammation and is mediated by adhesion molecules and cytokines [19]. ICAM-1 is an endothelial- and leukocyte-associated transmembrane protein long known for its importance in stabilizing cell-cell interactions and facilitating leukocyte endothelial transmigration [20]. More recently, ICAM-1 has been characterized as a site for the cellular entry of human rhinovirus [21]. Because of these associations with immune responses, many researchers have hypothesized that ICAM-1 could function in signal transduction. Earlier studies showed that ICAM-1 gene is highly expressed in pulmonary fibroblasts of COPD patients [22]. In addition, blocking pulmonary ICAM-1 expression ameliorates lung injury in established diet-induced pancreatitis [23]. Thus, adhesion molecules play a key role in regulating inflammation in respiratory disorders (Figure 1).

### 2.2. Cytosolic** **Phospholipase**** *A*_2_

There are three forms of PLA_2_ in mammalian cells [24]. The first class of PLA_2_ is secretary PLA_2_ (sPLA_2_) that is expressed in a variety of cell types [25] and it has no preference for AA at *sn*-2 position, requires millimolar amounts of Ca^2+^ for activity and is sensitive to sulfhydryl reducing agents, such as dithiothreitol (DTT), and is resistant to heat or acid conditions [25]. The second class of PLA_2_ is calcium-independent PLA_2_ (iPLA_2_) that does not require Ca^2+^ for catalytic activity. iPLA_2_ prefers plasmalogen substrates and does not appear to have a preference for the type of fatty acid at the *sn*-2 position [26]. The third class is the novel, high molecular weight (85 kDa) cytosolic PLA_2_ (cPLA_2_). cPLA_2_ enzymes catalyze the hydrolysis of the *sn*-2 position of membrane glycerophospholipids, leading to production of free fatty acids and lysophospholipids. This reaction is of particular importance if the esterified fatty acid is arachidonic acid (AA) [24], which is converted by downstream metabolic enzymes to various bioactive lipophilic compounds called eicosanoids, including prostaglandins (PGs) and leukotrienes (LTs) [24]. PLA_2_ could be the initial and rate-limiting enzyme in this conversion. The increase in cPLA_2_ activation and expression following external stimuli, including proinflammatory cytokines, growth factors, and microbial toxin, is often observed in several systems [27]. The implication of cPLA_2_ in inflammatory diseases has been confirmed by that the airway anaphylactic response in the cPLA_2_ knockout mice is markedly reduced compared with that in the wild-type mice [28]. Moreover, cPLA_2_-deficient mice have provided the most definitive evidence for the central role of cPLA_2_ in eicosanoid [29] as well as in the pathogenesis of several inflammatory diseases, such as acute respiratory distress syndrome (ARDS) due to bacterial sepsis [30, 31]. These studies have demonstrated that there was a reduction in the bronchial lumen and alveolar thickening in the control mice that was remarkably absent in the cPLA_2_ knockout mice. This outcome also appeared in 5-lipoxygenase (LO)-knockout mice and mice with PGD_2_ receptor deficiency [32]. Thus, cPLA_2_ seems to function as a crucial upstream regulator of the production of eicosanoids for airway resistance during allergic inflammation and is correlated to the process of asthma (Figure 1). The inhibition of cPLA_2_-mediated pathways may also provide a therapeutic approach to airway and lung injury.

### 2.3. Cyclooxygenase-2

COX metabolites have diverse effects in the lung and are known to modify airway tone as well as inflammatory responses [3]. Three isoforms of COX have been identified [24]. COX-1 is constitutively expressed in most tissues and considered to be the “housekeeping” isoform that produces PGs which are required for maintenance of normal cell and organ function. In contrast, COX-2 is primarily an inducible isoform whose expression can be upregulated in many cell types by cytokines, mitogens, and endotoxin [3, 4]. It is highly expressed in inflamed tissues and believed to produce PGs involved in inflammatory processes [33]. COX-2 has multiple transcriptional regulatory sequences in its promoter region, including a TATA box, an NF-IL6 motif, two AP-2 sites, three Sp1 sites, two NF-*κ*B sites, a CRE motif, and an E-box [34]. COX-2 gene expression can be induced by multiple cytokines and growth factors, via activation of transcriptional regulatory proteins that act on these promoter sites [35]. Thus, COX-2 appears to be the primary COX controlling PGE_2_ synthesis in response to inflammation (Figure 1). COX effects are widespread and extremely complex; however, studies in knockout mice for COX-1 versus COX-2 reveal sometimes overlapping, not altogether predictable roles for these two enzymes [24]. The levels of prostanoids in bronchoalveolar lavage fluid are increased in asthma, and several studies have found enhanced expression of both COX-1 and COX-2 in the airways of asthmatics [36, 37]. A recent research has renewed interest in the role of prostanoids in allergic airway disease. Moreover, the expression of COX-2 protein induced by *Lactobacillus rhamnosus* GG (LGG), endotoxin, and lipoteichoic acid (LTA) in T84 epithelial cells [38]. The presence of COX-3 mRNA transcript, with a size of approximately 5.2 kb, was subsequently confirmed in human cells; COX-3 was in highest concentrations in the cerebral cortex and heart tissue [24]. The regulation of COX-3 transcription appears to be identical to that of COX-1. COX-3 is similar to COX-1 and COX-2 in terms of structure and enzymatic function. However, the retention of intron 1 in COX-3 seems to slow its enzymatic activity in comparison to COX-1 and COX-2. Thus, the inhibition of COX-2-mediated inflammatory pathway may provide a therapeutic approach to respiratory diseases. 

### 2.4. Matrix Metalloproteinase-9

 MMPs are proteolytic enzymes that are able to degrade extracellular matrix (ECM) components and, thus, play a role in cell migration and tissue remodeling. Moreover, they can splice and (in)activate cytokines and chemokines, thereby influencing the recruitment and function of inflammatory cells [39]. To date, 24 MMPs have been identified in mammals; cellular sources include inflammatory, stromal, and epithelial cells. Some MMPs are anchored to the cell surface, whereas others are secreted into the extracellular space. They are released as inactive proenzymes and are activated by proteolytic cleavage of the N-terminal domain. Most MMPs are constitutively secreted once they become translated [40]. In gelatinase subfamily of MMPs (MMP-2 and MMP-9), the catalytic domain that includes the Zn^2+^ binding site also contains repeats of fibronectin motifs allowing the ability to bind gelatin, their major substrate. Patients with asthma have an increased gelatinolytic activity linked to MMP-2 and MMP-9 and higher levels of tissue inhibitor of metalloproteinase-1 (TIMP-1; a natural inhibitor of MMPs) in their sputum [41]. The activated form of MMP-9 (85 kDa) was found in the sputum from 60% of asthmatics, but was absent from that of control subjects. Although less frequently detectable than pro-MMP-9 (pro-MMPs are catalytically inactive and are activated into the active MMP after cleaving of the prodomain), pro-MMP-2 (72 kDa) was also found more frequently in asthmatics (50%) than in control subjects (5%). In addition, patients with COPD have an increased gelatinolytic activity in sputum linked to MMP-2 and MMP-9 [41]. In smokers with emphysema, MMP-8 and MMP-9 levels in bronchoalveolar lavage (BAL) fluid were significantly higher than in smokers without emphysema [42]. *In vitro* cultured human airway smooth cells and A549 cells, TNF-*α* and IL-1*β* induce MMP-9 expression and cell migration [2, 43, 44] via various signaling pathways, such as PKC, MAPKs, NF-*κ*B, and AP-1. Thus, MMPs and their inhibitors (TIMPs) play multiple functions in physiological processes and interact with many other mediators regulating inflammatory processes, cell behavior, and angiogenesis. These mediators are implicated in many intricate loops of reciprocal interactions rendering the understanding of the role of MMPs in regulatory processes difficult. In many respiratory diseases, MMPs are overexpressed or oversecreted leading to both deregulation of physiological homeostatic processes and ECM degradation and disorganization (Figure 1).

## 3. Inflammatory Signalings Involved in the Respiratory Diseases

### 3.1. Protein** **Kinase** **C

**Figure 2:**
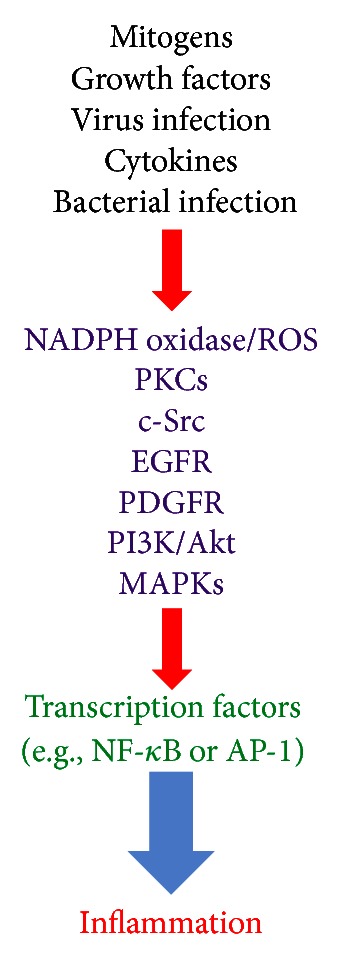
The signaling pathways in airway and pulmonary inflammation. Various stimuli, such as mitogens, growth factors, virus infection, cytokines, and bacterial infection, can induce inflammation via the NADPH oxidase/ROS, PKCs, c-Src, EGFR, PDGFR, PI3K/Akt, and MAPKs pathways and the transcription factors, such as NF-*κ*B and AP-1 in lung resident cells.

PKCs are important in many cellular responses in the lung, including permeability, contraction, migration, hypertrophy, proliferation, apoptosis, and secretion [45]. PKC is a family of serine/threonine kinases characterized by at least eleven different isotypes. PKC isotypes are differentially regulated by calcium (Ca^2+^), diacylglycerol, and phospholipids and differ in structure, expression, intracellular localization, substrate utilization, and mechanisms of activation [45]. The PKC isotypes are subdivided into three groups: the classical, novel, and atypical. This subdivision is based on the structural and functional differences in the conserved domains C1–C4. The classical PKC*α*, PKC*β*I/II, and PKC*γ* isotypes are Ca^2+^ and diacylglycerol dependent [46]. The novel PKC*ε*, PKC*δ*, PKC*η*, PKC*θ*, and PKC*μ* isotypes contain C2 domains that lack Ca^2+^-binding ability but still retain functional C1A and C1B domains that can bind the endogenous diacylglycerol and exogenous phorbol esters [47]. The atypical PKC*ι*, PKC*λ*, and PKC*ζ* isotypes lack a functional C2 domain and contain a single C1 domain that lacks the ability to bind diacylglycerol and phorbol esters. Therefore, the mechanism of activation of the atypical PKC isotypes is both Ca^2+^ and diacylglycerol independent. PKC*ζ* and PKC*λ* have been implicated in signaling through lipid metabolites including phosphatidylinositol 3-phosphates [47]. Moreover, PKCs are important signaling intermediates in chronic airway diseases like asthma and COPD. PKCs have been implicated in airway inflammation, bronchospasm, and mucous production [46]. Resident airway epithelial cells produce proinflammatory mediators under the regulation of PKC*δ* [48]. Increased PKC*δ* activity increases NF-*κ*B-dependent proinflammatory cytokine generation in human airway epithelial cells, while expression of a dominant negative PKC*δ* mutant has inhibiting effects. In human airway smooth muscle cells, PKC*α*, *β*I, *δ*, *ε*, *μ*, *γ*, and *ζ* are found in the cytosol and *β*II in the membrane under basal conditions [49]. The proinflammatory neuropeptide bradykinin (BK) causes activation of PKC*α*, *β*I, *δ*, and *ε* when applied to airway smooth muscle cells. BK also induces COX-2 protein expression and PGE_2_ accumulation in human airway smooth muscle cells via a PKC*ε*-dependent signaling. PKC*α* is increased in the lungs of patients with COPD and is thought to be important in the hypertrophy and proliferation of airway smooth muscle cells [46]. PKC*ζ* activity is also increased in proliferating human airway smooth muscle cells. On the other hand, PKC is important in mediating the effects of proinflammatory cytokines by phosphorylating cPLA_2_ leading to the release of AA from phospholipids with subsequent production of bioactive eicosanoids in activated cells [50]. PKC is a key regulator of fibrosis in human pulmonary interstitial fibroblasts. At least three PKCs are expressed in interstitial fibroblasts, including PKC*α*, *δ*, and *ε* [46]. Activation of PKC*α* causes decreased collagen expression via the extracellular signal-regulated kinase kinase (MEK)/ERK signaling cascade, a response that is opposed by PKC*ε* [51]. Selected PKCs are activated by LPS, leading to the production of the proinflammatory cytokines, such as TNF-*α*, IL-*β*, and IL-6 [52]. In addition, thrombin causes an increase in cytosolic [Ca^2+^] and activation of selected PKCs [53]. TNF-*α* has been shown to induce MMP-9 expression via a PKC*α*-dependent pathway in A549 cells [2]. Taken together, these studies indicated that PKCs play a critical role in mediating inflammation and respiratory diseases (Figure 2). Because multiple signaling pathways contribute to the key cellular responses important in lung biology, therapeutic strategies targeting PKCs may be more effective if combined with inhibitors of other pathways for additive or synergistic effect. Mechanisms that regulate PKC activity, including phosphorylation and interaction with isozyme-specific binding proteins, are also potential therapeutic targets in the respiratory diseases.

### 3.2. NADPH** **Oxidase/ROS

ROS are products of normal cellular metabolism and are known to act as second messengers. Under physiological conditions, ROS participate in maintenance of cellular “redox homeostasis” to protect cells against oxidative stress. In addition, regulation of redox state is important for cell activation, viability, proliferation, and organ function. However, overproduction of ROS, most frequently due to excessive stimulation of either reduced NADPH by proinflammatory cytokines or the mitochondrial electron transport chain and xanthine oxidase, results in oxidative stress. Oxidative stress is a deleterious process that leads to airway and lung damage and consequently to several respiratory inflammatory diseases/injuries [8]. ROS are intracellularly generated from several sources, including mitochondrial respiration, cytochrome P450, the NADPH oxidase system, and xanthine/xanthine oxidase [54]. However, the major ROS generating enzyme is NADPH oxidase, a membrane-bound multicomponent enzyme complex that is present in phagocytes as well as nonphagocytic cells [55]. ROS produced by NADPH oxidase have two major roles. First, superoxide produced by NADPH oxidase 2 is required for respiratory burst that occurs in phagocytes, leading to microbial killing. The second role of NADPH oxidase is associated with the regulation of cell signaling [55]. ROS derived from NADPH oxidase can specifically and reversibly react with proteins, altering their activity, localization, and half-life [8]. Activated phagocytic cells generate ROS via assembly and activation of the NADPH oxidase complex, which comprises membrane-associated flavocytochrome b_558_ (gp91^phox^) and p22^phox^ and various cytosolic cofactors (p47^phox^, p67^phox^, and p40^phox^, and the GTPase, Rac1) and mediates transmembrane electron transfer from the major cellular electron donor, NADPH, to reduce molecular O_2_ to superoxide anion (O_2_^•−^) and hydrogen peroxide (H_2_O_2_) [54]. A number of homologs of the main business end of NADPH oxidase, gp91^phox^, have been discovered, and mammalian systems are now known to contain seven NADPH oxidase homologs, comprising NADPH oxidase 1–5 (NADPH oxidase 2 being the new name for gp91^phox^) and two larger dual oxidases, DUOX1 and DUOX2, which are widely expressed in many cell types to mediate a variety of biological functions, such as cell mitosis, differentiation, migration, and immune regulation [56]. In *in vitro *studies, using macrophages, alveolar and bronchial epithelial cells, ROS have been shown to induce gene expression of inflammatory mediators, such as IL-1 and TNF-*α* [57, 58]. Patients with asthma demonstrate increased generation of ROS, such as superoxide anion, hydrogen peroxide, and hydroxyl radicals. Increased production of ROS has been demonstrated by many cell types within the lung in asthma, including macrophages, antigen-presenting cells (APCs), neutrophils, and eosinophils [59]. Excessive production of ROS correlates with the degree of airway hyperresponsiveness, as quantified by methacholine challenge. In addition, oxidative stress also contributes to a proteinase-antiproteinase imbalance, both by inactivating antiproteinases, such as *α*1-antitrypsin and secretory leukocyte proteinase inhibitor, and by activating proteinases, such as MMPs [8]. On the other hand, oxidants also promote inflammation by activating NF-*κ*B or AP-1, which orchestrates the expression of multiple inflammatory genes recognized to be important in COPD, such as TNF-*α*. Recently, phagocytic NADPH oxidase-ROS signaling has been shown to play a critical role in promoting TNF-*α*-induced, NF-*κ*B-dependent acute inflammatory responses, and tissue injury specifically in the lungs, which is effected by preferential leukocyte infiltration [8]. Thus, oxidative stress plays a critical role in inflammatory responses in airway and lung diseases via the upregulation of redox-sensitive transcription factors (such as AP-1 or NF-*κ*B) and thereby proinflammatory genes (such as MMP-9, VCAM-1, ICAM-1, COX-2, or cPLA_2_) expression. Inflammation itself results in oxidative stress in the airways and lungs. Taken together, NADPH oxidase/ROS play a critical role during development of airway and lung diseases (Figure 2).

### 3.3. PI3K/Akt

The PI3K family are central signaling elements in a diverse array of cellular functions, including growth, proliferation, migration, and survival. It is, therefore, understandable that dysregulation of PI3K has been implicated in the induction and/or progression of a variety of disease states, including those of the respiratory tract, ranging from asthma to cancer [60]. PI3Ks have been divided into three classes according to theirstructure and lipid substrate specificity [61]. The most extensively investigated are class I PI3Ks. Type I PI3Ks are activated by cell surface receptors, such as growth factors, insulin, and G-protein-coupled receptors (GPCRs). Class II PI3Ks comprised *α*, *β*, and *γ* isoforms, which are characterized by the presence of a C2 domain at the C terminus. They predominantly use phosphatidylinositol and phosphatidylinositol 4-phosphate [PI(4)P] as substrates. The class III PI3Ks only use phosphatidylinositol as a substrate. Class I PI3Ks are further divided into class IA and class IBPI3Ks. Structurally, PI3Ks IA exist as heterodimeric complexes in which a catalytic p110 subunit (designated as *α*, *β*, or *γ*) is in association with a particular regulatory subunit (designated as p85, p55, and p50) [61]. Importantly, PI3K IA signals downstream of receptor tyrosine kinase and Ras. The single class PI3K IB consists of the p110*γ* catalytic subunit complexed to the p101 regulatory subunit and signals downstream of GPCRs and Ras, which is activated by *βγ* subunits from GPCRs, such as the receptors for chemokines [61]. Despite limitations in selectivity, the two commercially available PI3K inhibitors, wortmannin and LY294002, have contributed greatly to our understanding of the biological role of PI3K in lung inflammation [62]. Moreover, previous study indicated that intratracheal administration of LY294002 significantly reduced ovalbumin-(OVA-) induced increases in total cell counts, eosinophil counts, and IL-5, IL-13, and CCL11 (eotaxin) levels in BAL fluid and dramatically inhibited OVA-induced tissue eosinophilia and airway mucus production [63]. This study confirmed that LY294002 markedly attenuated OVA-induced serine phosphorylation of Akt, a direct downstream substrate of PI3K. In addition, other studies also showed that LY294002 and wortmannin attenuated eosinophilic airway inflammation and airway hyperresponsiveness in a murine asthma model [64]. Thus, PI3K inhibition was indicated to have therapeutic potential for the treatment of asthmatic airway inflammation. On the other hand, ROS induction is accompanied by activation of PI3K. LY294002 was shown to reduce chemokine-induced ROS generation in phagocytes, which was further confirmed by studies using PI3K knockout mice [62]. It was also reported that serum withdrawal (SW) killed human U937 blood cells by elevating cellular ROS levels, which occurred through PI3K activation [65]. Thus, PI3K family plays a prominent role in various airway and lung inflammation (Figure 2). Moreover, inhibitors of PI3K/Akt may prove to be useful novel therapies in the treatment of respiratory diseases. 

### 3.4. Src** **Family** **Kinases

SFKs are signaling enzymes that have long been recognized to regulate critical cellular processes, such as proliferation, survival, migration, and metastasis [8]. Src protein tyrosine kinase (PTK) family is categorized into nonreceptor tyrosine kinases and consists of nine members. Src, Fyn, Yes, and Yrk are ubiquitously expressed, whereas Blk, Fgr, Hck, Lck, and Lyn are expressed in more restricted patterns [66]. So far, Yrk has been detected only in chicken [66]. Src PTK family members are activated in response to the stimulation of a variety of cell surface receptors, such as tyrosine kinase receptors, integrin receptors, and G protein-coupledreceptors, and by cellular stress [66]. Src PTKs can also regulate the functional activity of these receptors. Moreover, we reported that TNF-*α* or IL-1*β* induces VCAM-1 and ICAM-1 expression via a c-Src-dependent pathway in human airway smooth muscle cells [8]. In addition, c-Src has been shown to regulate COX-2/PGE_2_/IL-6-dependent airway inflammation via NADPH oxidase/ROS [67]. In human lung epithelial cells, in addition to activating NF-*κ*B-inducing kinase (NIK) via TRAF2, TNF-*α* could activate c-Src through PKC. Systemic inhibition of these kinases using specific small molecule inhibitors for Src PTKs (either PP2 or SU-6656) significantly attenuated LPS-induced lung injury and capillary permeability and reduced LPS-dependent cytokine and chemokine levels in the lung and the serum [68]. Thus, the role of Src family PTKs in inflammatory responses is a rising area of research (Figure 2). However, application of small chemical inhibitors to effectively and specifically block Src PTKs could have a great clinical implication for airway and lung diseases with inflammatory responses as underlying mechanisms.

### 3.5. Growth** **Factor** **Tyrosine** **Kinase** **Receptors

Cell-surface tyrosine kinases receptors play pivotal roles in development, tissue repair, and normal cellular homeostasis. Aberrant expression or signaling patterns of these receptors have also been linked to the progression of a diversity of diseases, including asthma. Two major families of tyrosine kinases receptors, the epidermal growth factor receptor (EGFR) and platelet-derived growth factor receptor (PDGFR) families, have received a great deal of attention as potentially therapeutic targets for respiratory diseases, as these receptors have been shown to play key roles in chronic tissue remodeling in asthma, bronchitis, and pulmonary fibrosis. The EGFR system on epithelial cells and underlying mesenchymal cells (fibroblasts, myofibroblasts, and smooth muscle cells) drives numerous phenotypic changes during the progression of these pulmonary diseases, including mesenchymal cell hyperplasia, differentiation, and ECM production. The PDGFR system, located primarily on mesenchymal cells, transduces signals for cell survival, growth, and chemotaxis. The variety of EGFR and PDGFR ligands produced by the airway epithelium or adjacent mesenchymal cells allows for intimate epithelial-mesenchymal cell communication. In humans, the airway epithelium expresses EGFR ligands constitutively, including EGF, TGF-*α*, HB-EGF, amphiregulin, heregulin, and betacellulin. Expression of several EGFR ligands has also been investigated in diseases, such as COPD and asthma. Kohri et al. showed that *P. aeruginosa* bacterial supernatant induces mucin production in human airway epithelial cells (NCI-H292) via EGFR activation [69]. Multiple studies have also reported that stimulation of airway epithelial cells by LPS induces the secretion of IL-8 via a cellular cascade involving a TLR4/myeloid differentiation primary response gene (MyD)88/NF-*κ*B-dependent pathway [70]. In addition, ROS have been shown to stimulate PDGFR*α* activation via c-Src family kinases [8]. There is accumulating evidence that PKC-dependent phosphorylation of p47^phox^ is essential for PDGF-stimulated ROS generation, which is important for PDGF-induced MAPKs activation [8]. Taken together, these studies suggest that growth factor tyrosine kinase receptors may also play a key role in mediating expression of inflammatory genes (Figure 2). 

### 3.6. Mitogen-Activated** **Protein** **Kinases

MAPKs are important components of signaling modules activated by neurotransmitters, cytokines, and growth factors, as well as chemical and mechanical stressors. In the airway, these external signals produce acute responses that modify smooth muscle contraction and may also induce chronic responses that modify airway structure [71]. Both acute and chronic events in airway remodeling result from altered expression of multiple genes encoding protein mediators of cell-cell signaling, ECM remodeling, cell cycle control, and intracellular signaling pathways [72]. In mammals, three groups of MAPKs have been identified: the extracellular signal-regulated protein kinases (ERKs), the c-Jun NH_2_-terminal kinases (JNKs), and the p38 MAPK. ERK is activated by diverse stimuli, including growth factors and cytokines [73]. The p38 MAPK is activated by cellular stresses, including UV radiation, LPS, growth factors, and cytokines. The JNK is activated by many of the same stimuli that activate p38 MAPK, such as cellular stresses and numerous cytokines. Thus, the inhibition of MAPKs activity via pharmacological or genetic approaches blocks allergic inflammation of airways. Moreover, asthmatic patients demonstrated increased immunostaining for phospho (p)-ERK, p-p38 MAPK, and p-JNK [72]. p-ERK staining was observed especially in airway epithelium and smooth muscle cells. The phosphorylation of p38 MAPK was primarily observed in the basal layer of the columnar epithelium. It is likely that p38 MAPK drives basal metabolic processes for this particular cell type. There was significant correlation between clinical severity of asthma and intensity of immunostaining for p-ERK and p-p38 MAPK and between p-ERK and the number of tissue eosinophils and neutrophils in the airways. p-JNK primarily stained airway smooth muscle cells. Early studies of p38 MAPK demonstrated that IL-1*β* and TNF-*α* activate the p38 MAPK in monocytes [74]. Furthermore, inhibition of the p38 MAPK pathway was shown to exert anti-inflammatory effects through inhibition of IL-1*β*, IL-6, and TNF-*α* expression [75]. In airway smooth muscle cells, there is solid evidence that both ERK and p38 MAPK pathways contribute to IL-1*β*-induced COX-2 expression and PGE_2_ synthesis [76, 77]. In a mouse model of chronic lung inflammation (allergic inflammation), significant inhibition of TNF-*α*, IL-4, IL-13, and RANTES (regulated on activation, normal T-cell expressed and secreted) in lung homogenates was observed with JNK inhibitor, SP600125 [78]. In addition, we also found that LTA or IL-1*β* could induce cPLA_2_, COX-2, or MMP-9 in human airway smooth muscle cells or A549 cells [3, 44]. Therefore, MAPKs play an important role in mediating airway and lung inflammation (Figure 2). 

### 3.7. NF-*κ*B

NF-*κ*B is viewed as a master regulator of inflammatory responses because it plays an essential role in the evolution as well as the resolution phase of inflammation. NF-*κ*B controls a wide spectrum of biological effects ranging from immune and stress-induced responses to cell fate decisions such as proliferation, differentiation, tumorigenesis, apoptosis, and tissue remodeling [8]. NF-*κ*B usually exists as a heterodimeric complex of p50 and p65/RelA subunits. In unstimulated cells, NF-*κ*B is found in the cytoplasm as an inactive non-DNA-binding form, associated with an inhibitor protein called inhibitory *κ*B (I*κ*B) which masks the nuclear translocation signal and so prevents NF-*κ*B from entering the nucleus. Upon cell stimulation with various NF-*κ*B inducers, I*κ*B*α* is rapidly phosphorylated on two serine residues, which targets the inhibitor protein for ubiquitination by the E3 ubiquitin-ligases (E3RSI*κ*B) and subsequent degradation by the 26S proteasome [79]. The released NF-*κ*B dimer can then be translocated into the nucleus and activate target genes by binding with high affinity to *κ*B elements in their promoters. NF-*κ*B is activated by numerous extracellular stimuli, including cytokines such as TNF-*α* and IL-1*β*, viruses and environmental particulates (PM10s), and oxidative stress [8]. Exogenous H_2_O_2_ also activated NF-*κ*B in a murine model of ROS-induced acute lung injury. Administration of OTC (L-2-oxothiazolidine-4-carboxylate) resulted in significant reduction of NF-*κ*B translocation into the nucleus and expression of adhesion molecules, chemokines, and cytokines [80]. Previous study demonstrated that NF-*κ*B activation occurred rapidly in the ovalbumin (OVA) model of allergic airways disease and that NF-*κ*B activation predominantly occurred in the epithelial cells of the conducting airways, in association with enhanced mRNA expression of NF-*κ*B-regulated chemokine genes, including MIP-2 and eotaxin [81]. A novel cyclin-dependent kinase inhibitor (BAI) has been shown to downregulate TNF-*α*-induced expression of cell adhesion molecules by inhibition of NF-*κ*B activation in human pulmonary epithelial cells [82]. Recently, we also demonstrated that overexpression of HO-1 protects against TNF-*α*-mediated airway inflammation by downregulation of TNFR1-dependent oxidative stress and NF-*κ*B activation [83]. Taken together, these results show that NF-*κ*B plays a key role in mediating the expression of inflammatory proteins in airway and lung inflammation and injury (Figure 2).

### 3.8. AP-1

AP-1 transcription factor typically consists of combinations of Jun (c-Jun, Jun B, Jun D) and Fos proteins (c-Fos, Fos B, Fra-1, Fra-2), which bind to the promoters of target genes. It was found to be responsible for the transcriptional activation of various genes that were activated by phorbol esters (such as PMA) via activation of PKC [84]. AP-1 may be activated via PKC and by various cytokines, including TNF-*α* and IL-1*β*, via several types of PTK and MAPKs, which themselves activate a cascade of intracellular kinases [85]. Certain signals rapidly increase the transcription of the fos gene, resulting in increased synthesis of Fos protein. Other signals lead to activation of kinases that phosphorylate c-Jun, resulting in increased activation. Specific Jun and Fos kinases are now recognized and may play a key role in the regulation of cellular responsiveness to cytokine signals. Recent studies showed that sirtuin 1 (SIRT1) directly interacted with c-Jun and repressed the transcriptional activity of AP-1, thus decreasing MMP-9 expression [86]. More recently, it was reported that SIRT1 decreased c-Fos/c-Jun acetylation induced by p300 and inhibited the transcriptional activity of AP-1 and subsequent COX-2 expression and PGE_2_ generation [87]. Thus, AP-1 may play a critical role in mediating expression of various inflammatory proteins. There is evidence for increased expression of c-Fos in epithelial cells in asthmatic airways [88], and many of the stimuli relevant to asthma that activate NF-*κ*B will also activate AP-1. Thus, AP-1 is also a key factor in respiratory diseases (Figure 2).

## 4. Therapeutic Implications

Kinase pathways have become recognized as key cellular signal transducers, and several protein kinase inhibitors are in development for the treatment of respiratory diseases. The pyridinylimidazole compounds, exemplified by SB203580, were originally prepared as inflammatory cytokine synthesis inhibitors that subsequently were found to be selective inhibitors of p38*α* and *β* MAPK [89]. SB203580 inhibits the catalytic activity of p38 MAPK by competitive binding inthe ATP pocket. These drugs inhibit many inflammatory cytokines, chemokines, and inflammatory enzymes [89]. SB203580 was shown to attenuate BAL TNF-*α* production in an ovalbumin challenged rat model of asthma [90] and SB2439063 reduced neutrophilia and mediator expression in rat COPD models [91]. In addition, a recent study also indicated that in acute and chronic animal models of asthma, SP600125 (a JNK inhibitor) reduces BAL accumulation of eosinophils and lymphocytes, cytokine release, serum IgE production, and smooth muscle proliferation after repeated allergen exposure [92]. Intratracheal administration of LY294002 reduced OVA-induced increases in total cell counts, eosinophil counts, and IL-5, IL-13, and CCL11 (eotaxin) levels in BAL fluid and dramatically inhibited OVA-induced tissue eosinophilia and airway mucus production [63]. Inhibition of SFKs using specific inhibitors for Src PTKs (either PP2 or SU-6656) attenuated LPS-induced lung injury and capillary permeability and reduced LPS-dependent cytokine and chemokine levels in the lung and the serum [68].

RNAi is the process of sequence-specific, post-transcriptional/transcriptional gene silencing through small interfering RNA (siRNA). RNAi is a popular method of controlling gene expression and has a potential in the development of drugs for several diseases, such as various types of cancer and viral infections. Gene therapy for asthma has already been developed and has demonstrated promising results in animal models [93]. Recent progress in delivering siRNA to the respiratory system has also improved the therapeutic feasibility of RNAi for asthma. IL-5 has been suggested to be involved in the development of airway hyperresponsiveness. Huang et al. indicated that siRNA against IL-5 decreases airway eosinophilia and hyperresponsiveness [93]. In the context of allergic immune responses, activation of STAT6 is pivotal for Th2-mediated IgE production and development of airway inflammation and hyperreactivity [94]. Moreover, STAT6 siRNA has been shown to inhibit allergic airway inflammation and hyperreactivity in mice [94].

Inflammatory airway and lung diseases are characterized by chronic inflammation and oxidant/antioxidant imbalance, a major cause of cell damage/injury. Numerous studies have shown the effectiveness of polyphenols in limiting the progression of chronic diseases. This is likely to occur, at least in part, because of the antioxidant capacity of these molecules, which extends from the availability of hydroxyl groups and the presence of conjugated double bonds. Resveratrol has been reported to increase antioxidant capacity and reduce various markers of oxidative stress [8]. Recently, Lee et al. indicated that resveratrol inhibits the activation of NF-*κ*B (p65) by TNF-*α* or PMA and reduces ATP-induced mucin secretion from cultured primary rat tracheal surface epithelial (RTSE) cells [95]. On the other hand, edaravone (3-methyl-1-phenyl-2-pyrazolin-5-one), a novel radical scavenger protects neurons by reducing endothelial injury and by ameliorating neuronal damage caused by brain ischemia. Treatment of edaravone decreases interstitial edema and inflammatory cell infiltration as well as prevents the process of pulmonary fibrosis [8]. In addition, reports of clinical benefit in airway and lung diseases for increased vitamins C and E and other dietary antioxidants have been varied. Morita et al. indicated that vitamin E treatment prior to injury largely prevents the increase in pulmonary permeability index and moderates the increase in lung lymph flow, increases the PaO_2_/FiO_2_ ratio, ameliorates both peak and pause airway pressure increases, and decreases plasma conjugated dienes and nitrotyrosine [96]. Erdosteine is a thiol antioxidant having mucoactive properties and the ability to reduce bacterial adhesiveness. This compound was introduced as a mucolytic agent for the treatment of chronic airway and pulmonary diseases. Erdosteine breaks the disulfide bonds of mucus glycoproteins, affecting the physical properties of the mucus, thus leading to increased cough clearance [97]. In addition, erdosteine has been reported to have antioxidant, anti-inflammatory, and antibacterial activity [97]. Negro et al. showed that erdosteine at a dose of 600 mg/day proved effective in significantly reducing ROS levels in peripheral blood of stable COPD patients who are current smokers, together with reduction in levels of some chemotactic proinflammatory cytokines (IL-6 and IL-8) in their bronchial secretions [98].

Recently, Greene and Gaughan represent new therapeutic targets and medicines that target specific microRNAs (miRNAs) and may have potential in the treatment of asthma [99]. miRNAs are regulatory RNAs that affect protein synthesis [99]. There have been a number of studies in the field of miRNA that implicate specific miRNAs in the pathophysiology of asthma. For example, studies using mouse models have identified miRNAs that are altered in response to allergen challenge. Certain miRNAs that are involved in the regulation of IL-13 and the TH2 response, key components of the asthmatic response, have been shown to be amenable to modulation by pre-miRs and anti-miRs. Other studies have identified miRNAs that are implicated in bronchial smooth muscle hyperresponsiveness and proliferation. Thus, developing miRNA-based medicines to treat the pulmonary manifestations of asthma could yield therapeutics with new properties that have the potential to treat both the inflammation and hyperresponsiveness associated with this disease.

## 5. Conclusions

**Figure 3:**
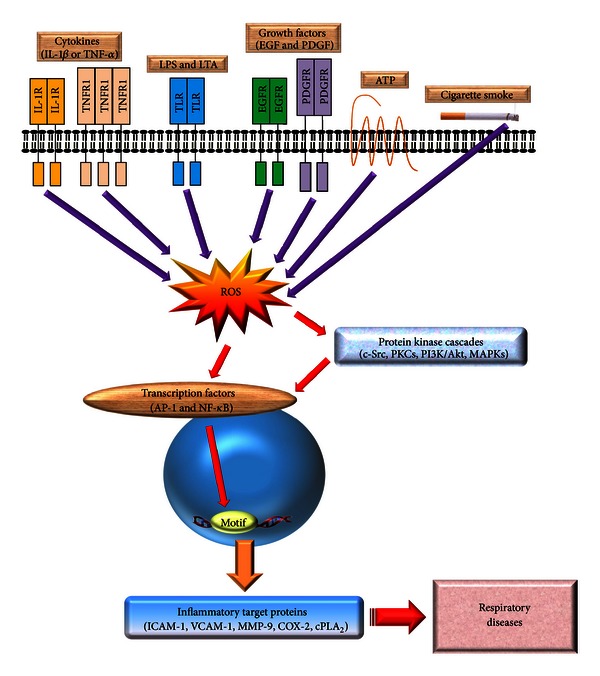
General overview of the inflammatory signaling pathways in respiratory diseases. Many of the known inflammatory target proteins, such as MMP-9, ICAM-1, VCAM-1, COX-2, and cPLA_2_, are associated with inflammatory signaling pathways induced by various stimuli, including TNF-*α*, IL-1*β*, ATP, cigarette smoke, LTA, or LPS. Moreover, the SFKs, PKCs, growth factor tyrosine kinase receptors, NADPH oxidase/ROS, PI3K/Akt, and MAPKs are components of signaling cascades that respond to extracellular stimuli by targeting transcription factors, such as NF-*κ*B and AP-1, resulting in the modulation of inflammatory gene expression associated with pulmonary diseases.

There is an increasing evidence that inflammatory proteins, such as VCAM-1, ICAM-1, cPLA_2_, COX-2, and MMP-9 are involved in the pathogenesis of respiratory diseases, such as asthma and COPD (Figure 3). Moreover, various inflammatory signaling pathways, including PKCs, NADPH oxidase/ROS, EGFR, PDGFR, c-Src, PI3K/Akt, MAPKs, AP-1, and NF-*κ*B, are involved in the regulation of these inflammatory proteins (Figure 3). Further exploration of the role of VCAM-1, ICAM-1, cPLA_2_, COX-2, and MMP-9 in these highly prevalent diseases is crucial to identify which are possible therapeutic targets. The development of new inhibitors that are highly specific but have no major adverse effects is essential. As targeted delivery of inflammatory proteins inhibitors directly to the airway and lung might result in fewer side effects, this option should also be explored. Although the use of inhibitors of inflammatory signaling pathways in the treatment of respiratory diseases seems very attractive, further studies are needed to identify the exact role of inflammatory signaling molecules in these diseases and to develop highly specific inhibitors.
